# Occurrence, Homologue Profiles and Risk Assessment of Short- and Medium-Chain Chlorinated Paraffins in Edible Vegetable Oils

**DOI:** 10.3390/foods14233988

**Published:** 2025-11-21

**Authors:** Yu Lu, Nan Wu, Lirong Gao, Lei Zhang, Tingting Zhou, Pei Cao, Jinyao Chen, Pingping Zhou

**Affiliations:** 1 West China School of Public Health and West China Fourth Hospital, Food Safety Monitoring and Risk Assessment Key Laboratory of Sichuan Province, Sichuan University, Chengdu 610041, China; luyu111112025@163.com; 2 NHC Key Laboratory of Food Safety Risk Assessment, China National Center for Food Safety Risk Assessment, Beijing 100022, China; 3 State Key Laboratory of Environmental Chemistry and Ecotoxicology, Research Center for Eco-Environmental Sciences, Chinese Academy of Sciences, Beijing 100085, China

**Keywords:** chlorinated paraffins (CPs), short-chain chlorinated paraffins (SCCPs), medium-chain chlorinated paraffins (MCCPs), dietary exposure, risk assessment, edible vegetable oils

## Abstract

Dietary intake is the major route of human exposure to fat-soluble and persistent chlorinated paraffins (CPs), which tend to accumulate in lipid-rich foods such as edible vegetable oils. This study investigated the levels of short-chain (SCCPs) and medium-chain chlorinated paraffins (MCCPs) in commercially available vegetable oils and assessed their potential health risks. The concentrations of SCCPs and MCCPs in 29 commercial edible vegetable oils were analyzed using comprehensive two-dimensional gas chromatography coupled with electron capture negative ionization mass spectrometry (GC × GC-ECNI-MS). Dietary exposure levels were estimated through probabilistic assessment integrating analytical results with dietary consumption data from the Chinese Total Diet Study (2017–2020). The margin of exposure (MOE) approach was employed for risk characterization. The average concentrations of SCCPs and MCCPs were 112 ng/g and 139 ng/g, respectively. The highest SCCP and MCCP concentration were found in sesame oil and peanut oil, respectively. Overall, MCCPs levels were generally higher than SCCPs. The estimated daily intakes (EDIs) of SCCPs and MCCPs were 56.06 and 73.63 ng/kg bw/d on average, with high consumers (P95) exposed to 180.91 and 230.49 ng/kg bw/d, respectively. Corresponding MOE at P95 were 1.27 × 10^4^ for SCCPs and 1.56 × 10^5^ for MCCPs. The current SCCPs and MCCPs dietary intake originated from edible vegetable oils did not pose a significant health risk. This study provides the first probabilistic exposure assessment of CPs in Chinese edible vegetable oils, offering current contamination profiles.

## 1. Introduction

Chlorinated paraffins (CPs) are synthetic mixtures of polychlorinated n-alkanes widely used as plasticizers, lubricants, sealants, metalworking fluids, and flame retardants [1]. Based on carbon chain length, CPs are subdivided into short-chain CPs (SCCPs, C_10-13_), medium-chain CPs (MCCPs, C_14-17_), and long-chain CPs (LCCPs, ≥C_18_) [2]. China is the world’s largest producer and consumer of CPs, with an annual production of up to 1.05 million tons in 2013. According to chlorine content, industrial products are commonly divided into three major types: CP-42, CP-52, and CP-70 [3]. Toxicological and epidemiological studies have shown that CPs exhibit multisystem toxicity, including hepatotoxicity, nephrotoxicity, developmental and neurotoxic effects, endocrine disruption, immunotoxicity, and reproductive toxicity [4,5]. Consequently, CPs have attracted considerable scientific and regulatory attention due to their potential risks to human health and environmental safety.

Dietary intake is the primary route of human exposure to CPs, accounting for approximately 90% of total exposure in non-occupational populations [6]. SCCPs and MCCPs are particularly significant, with food consumption contributing 50–100% and 70–100% of total human exposure, respectively [7]. Given the high lipophilicity of CPs, edible vegetable oils, as essential components of daily diets, serve as a major source of CPs contamination [8,9]. Market basket studies in China [10], Germany [11] and Korea [9] have consistently reported higher CP concentrations in fats and oils than in other food categories. Similarly, Nilsson et al. [12] reported that oil samples contained the highest SCCPs levels among various foods in Japan, while Cao et al. [8] found that edible oils accounted for approximately 32.2% of the total dietary SCCPs exposure in Beijing residents. Moreover, in certain regions of China, fried foods contribute a substantial share of total dietary SCCPs intake [13]. CPs are also detected in human blood, urine, and breast milk, with body burdens in the Chinese population exceeding those observed in other countries [14]. Although several studies have investigated the distributions and congener group profiles of SCCPs and MCCPs in edible vegetable oils and evaluated their dietary exposure levels, the majority have employed deterministic approaches. For example, Gao et al. [15] estimated that the mean dietary intakes of SCCPs and MCCPs from cooking oils among the general Chinese population were 8.83 and 6.09 μg/kg bw/day, respectively. Probabilistic assessments, which can better characterize the variability and uncertainty in dietary exposure, remain scarce. Thus, this study provides the first probabilistic exposure assessment of CPs in Chinese edible vegetable oils, offering current contamination profiles.

Moreover, SCCPs have been listed in Annex A of the Stockholm Convention since 2017 [16], and China has fully phased out the production, use, and import/export of SCCPs since 1 January 2024, under the List of Key Controlled New Pollutants (2023 Edition) [17]. Meanwhile, the inclusion of MCCPs in Annex A has been formally supported by the European Union in 2025 [18]. Given this evolving regulatory landscape, investigating the contamination levels of SCCPs and MCCPs in edible vegetable oils and evaluating their associated health risks are of great importance.

Therefore, this study aimed to: (1) present a current evaluation of the occurrence and congener group profiles of SCCPs and MCCPs in edible vegetable oils; (2) estimate the dietary exposure and the potential health risks of the Chinese population using a probabilistic assessment approach; and (3) evaluate the potential effects of recent regulatory measures on the contamination trends of SCCPs and MCCPs in edible vegetable oils.

## 2. Materials and Methods

### 2.1. Determination of SCCPs and MCCPs in Edible Vegetable Oils

#### 2.1.1. Materials and Reagents

The vegetable oil samples used in this study were provided by the China National Center for Food Safety Risk Assessment (CFSA). In 2023, we collected a total of 29 samples of six vegetable oil types commonly available on the Chinese market, including peanut, corn, soybean, sunflower, rapeseed, and sesame oils. All samples were regular refined oils purchased from retail markets, and they covered major commercial brands in China, such as Jinlongyu, Luhua, Fulinmen, and Xiwang. Further details on these samples can be found in Table S1.

Standard solutions of SCCPs (chlorine contents: 51.5%, 55.5%, 63.0%; 100 µg/mL, in cyclohexane) and MCCPs (chlorine contents: 42.0%, 52.0%, 57.0%; 100 µg/mL, in cyclohexane) were purchased from Dr. Ehrenstorfer (Augsburg, Germany). The reference material ^13^C_10_-trans-chlordane (100 µg/mL, in nonane; Cambridge Isotope Laboratories, Tewksbury, MA, USA) was used as the cleanup standard, while ε-hexachlorocyclohexane solution (ε-HCH, 10 µg/mL, in cyclohexane; Dr. Ehrenstorfer, Augsburg, Germany) served as the injection standard.

Pesticide-grade n-hexane, acetone, cyclohexane, and dichloromethane were purchased from J.T. Baker (Phillipsburg, NJ, USA). Analytical-grade methanol, acetone, and dichloromethane (Sinopharm Chemical Reagent Co., Ltd., Shanghai, China) were used for rinse glassware prior to analysis. Diatomaceous earth was supplied by Thermo Fisher Scientific (Waltham, MA, USA). All adsorbents were pre-cleaned in a muffle furnace prior to use. Silica gel (63–100 μm; Merck KGaA, Darmstadt, Germany) and Florisil (60–100 mesh; Merck KGaA, Darmstadt, Germany) were activated by heating at 550 °C for 6.5 h and 12 h, respectively. Anhydrous sodium sulfate (analytical reagent grade; Comeo Chemical Reagent Co., Tianjin, China) was activated by heating at 650 °C for 6.5 h. Acidic silica gel (44%, *w*/*w*) was slowly adding 43.8 mL of 98% sulfuric acid dropwise to 100 g of activated silica gel under continuous stirring, followed by shaking for at least 2 h to ensure homogeneity [19].

#### 2.1.2. Instruments

Analysis was performed using comprehensive two-dimensional gas chromatography with electron capture negative ionization mass spectrometry (GC × GC-ECNI-MS; Agilent Technologies, Santa Clara, CA, USA) equipped with a single-jet cryogenic thermal modulator (ZX2004, Zoex, Houston, TX, USA). Chromatographic separation was achieved using a DB-5MS column (30 m × 0.25 mm × 0.25 μm; Agilent Technologies, Santa Clara, CA, USA) in the first dimension and a BPX-50 column (1 m × 0.10 mm × 0.10 μm; SGE, Melbourne, VIC, Australia) in the second dimension.

#### 2.1.3. Extraction Protocol

An amount of 2.00 g of vegetable oil was extracted with 70 mL n-hexane/dichloromethane (1:1, *v*/*v*) and spiked with 2.5 ng ^13^C_10_-trans-chlordane. Lipids were removed with acidified silica gel, and extracts were concentrated and purified on a multilayer silica column (Florisil, activated silica, acidified silica, anhydrous sodium sulfate). The final eluate was concentrated, spiked with 2.5 ng ε-HCH as internal standard, adjusted to 50 μL with cyclohexane, and analyzed. Detailed extraction and purification procedures are provided in Supplementary Text S1.

#### 2.1.4. Instrumental Analysis

Analyses were performed using a comprehensive two-dimensional gas chromatograph coupled with an electron capture negative chemical ionization mass spectrometer (GC× GC-ECNI-MS; Agilent Technologies, Santa Clara, CA, USA). A DB-5MS column (30 m × 0.25 mm × 0.25 μm; Agilent Technologies, Santa Clara, CA, USA) was used as the first-dimension column, and a BPX-50 column (1 m × 0.1 mm × 0.1 μm; ; SGE, Melbourne, VIC, Australia) as the second-dimension column. The injector temperature was set at 280 °C. The oven temperature program started at 60 °C, increased at 10 °C/min to 180 °C, then at 1.5 °C/min to 310 °C, and was held for 5 min. The injection volume was 1 μL in splitless mode under constant flow, with a modulation period of 7 s. Mass spectrometric detection was conducted in ECNI mode with a resolution of ≥5000, an ion source temperature of 200 °C, and data acquired in selected ion monitoring (SIM) mode.

#### 2.1.5. Quality Assurance and Quality Control (QA/QC)

Before analysis, all glassware was sequentially rinsed three times with ultrapure water, methanol, acetone, and dichloromethane. Procedural blanks (one per ten samples) were analyzed to monitor background contamination. These procedural blanks were extracted and analyzed in the same way as the samples. The mean concentrations of SCCPs and MCCPs in the blanks were 25 ng/g and 10 ng/g, respectively. The recovery of the surrogate standard (^13^C-10-trans-chlordane), calculated relative to ε-HCH, ranged from 59% to 106% (mean: 81%). For every fifteen samples, one duplicate sample was randomly selected for parallel analysis, and the relative standard deviation (RSD) was below 15%. The limits of detection (LODs) were calculated by tripling the standard deviation of the average levels of three blanks samples. The LODs for SCCPs and MCCPs were 32 ng/g and 16 ng/g, respectively. A Total Ion Chromatogram (TIC) of a Vegetable Oil Sample is shown as a representative example in Figure S1. Representative ECNI-MS spectra used for congener identification (C10Cl6 and C14Cl7) are provided in the Supplementary Material (Figures S2 and S3).

### 2.2. Dietary Consumption Data of Vegetable Oils in China

Vegetable oil consumption data were obtained from the Chinese National Food Consumption Survey conducted by CFSA during 2017–2020, which used a multistage stratified cluster random sampling proportional to population size. The survey design and adult oil consumption results have been previously reported [20]. In this study, a total of 37,649 individuals covering all age groups were included in the analysis. Oils were categorized into six major edible oil types, including peanut, corn, soybean, sunflower, rapeseed, and sesame. After excluding participants with missing demographic information, missing cooking oil data, or implausible intake values (<P1 or >P99), 37,649 individuals were included in the analysis.

### 2.3. Chlorinated Paraffin Exposure Assessment

#### 2.3.1. Probabilistic Assessment Method

A probabilistic assessment approach based on Monte Carlo simulation was applied to estimate the dietary exposure risks of SCCPs and MCCPs from six types of vegetable oils among general and high-consumption populations. The model integrated body weight and vegetable oil consumption data from 37,649 individuals, along with the measured concentrations of SCCPs and MCCPs in the six oil types. Concentrations below the LOD were replaced with one-half the LOD. All input variables, including consumption and concentration data, were fitted to appropriate probability distributions using the RiskCumul probabilistic fitting approach prior to simulation. A total of 10,000 iterations were conducted using @RISK 8.8.1 software. Dietary exposure was estimated according to the following Equation (1) [21]:

(1) $$\mathrm{EDI}=\sum_{i\;=\;1}^{n}\frac{P_{i}\times C_{i}}{{\mathrm{Bw}}_{i}}$$

where EDI is the daily intake of SCCPs and MCCPs (ng/kg bw/d); $P_{i}$ represents the consumption of the *i*-th vegetable oil (g/d); $C_{i}$ is the concentration of SCCPs and MCCPs in the corresponding oil sample (ng/g); and ${\mathrm{Bw}}_{i}$ denotes the individual body weight (kg).

#### 2.3.2. Risk Characterization

The MOE approach was applied to assess the potential health risks associated with SCCPs and MCCPs intake from edible vegetable oils [22]. The MOE was calculated according to the following Equation (2):

(2) $$MOE=\frac{{BMDL}_{10}}{EDI}$$

where BMDL represents the benchmark dose lower confidence limit, and EDI denotes the estimated daily intake. According to the European Food Safety Authority (EFSA), BMDL_10_ values of 2.3 and 36 mg/kg bw/d were selected for SCCPs and MCCPs, based on increased nephropathy incidence in male rats and elevated relative kidney weights in both sexes, respectively [23]. An MOE value below 1000 indicates a potential health concern requiring further attention.

## 3. Results

### 3.1. Concentrations of SCCPs and MCCPs in Edible Vegetable Oils

**Figure 1:**
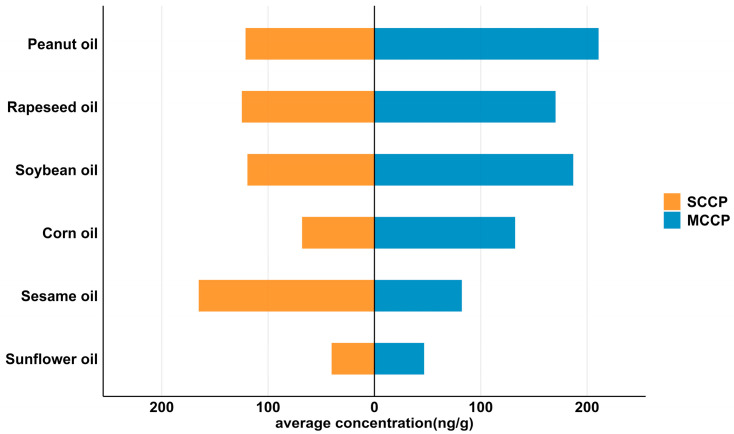
Average concentration of SCCPs and MCCPs in different vegetable oils (ng/g; *n* = 29).

CPs were detected in nearly all analyzed vegetable oil samples. Among them, SCCPs were detected in 27 samples, with concentrations ranging from non-detected (ND) to 283 ng/g (mean: 112 ng/g), while MCCPs ranged from 17 to 341 ng/g (mean: 139 ng/g). Overall, MCCP concentrations were generally higher than those of SCCPs. As shown in Figure 1 and Table S2, CP concentrations varied across different oil types. The mean SCCP and MCCP concentrations in sunflower, peanut, soybean, sesame, rapeseed, and corn oils were 54 and 47, 121 and 211, 119 and 187, 165 and 82, 125 and 170, and 79 and 132 ng/g, respectively. The highest SCCPs level was observed in sesame oil, while peanut oil exhibited the highest MCCP concentration.

### 3.2. Congener Group Profiles of SCCPs and MCCPs in Different Vegetable Oils

**Figure 2:**
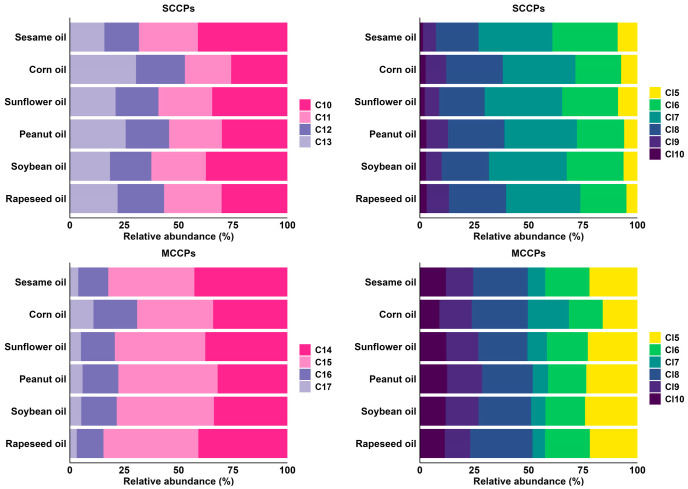
Congener group abundance profiles of SCCPs and MCCPs in different types of vegetable oil (*n* = 29).

As shown in Figure 2, the congener group profiles of SCCPs and MCCPs were examined in six types of vegetable oils commonly consumed in China. Detailed concentration data are provided in Tables S3 and S4. For SCCPs, C_10_Cl_6-7_ were the predominant congeners. By carbon chain length, C_10_-CP was the most abundant congener across all oil samples, accounting for an average of 34%, followed by C_11_-CP (25%), C_13_-CP (21%), and C_12_-CP (19%). Corn oil was an exception, where C_13_-CP was dominant (31%), whereas in the other oils, C_10_-CP remained the most abundant (30–41%). Regarding chlorination patterns, C_l6_-CP, C_l7_-CP, and C_l8_-CP dominated all samples, together accounting for 65% of total SCCP concentrations, with individual oil profiles ranging from 19% to 35%. Among them, C_l7_-CP was the predominant congener in all oil types.

The congener group profiles of MCCPs in vegetable oils were characterized by the predominance of C_15_Cl_6-8_. By carbon chain length, the average contributions of C_14_-, C_15_-, C_16_-, and C_17_-CPs were 36%, 42%, 16%, and 6%, respectively. Sesame oil was an exception, with C_14_-CP (43%) as the dominant congener, whereas other oils were mainly composed of C_15_-CP (34–45%) and C_16_-CP as a secondary congener (12–20%). Regarding chlorination, Cl_5_-, Cl_6_-, and Cl_8_-CPs dominated all samples, collectively accounting for 65% of MCCPs. Cl_8_-CP was the major chlorinated congener (22–28%), followed by Cl_5_-CP (15–24%) and Cl_6_-CP (15–20%).

### 3.3. Vegetable Oil Consumption

**Figure 3:**
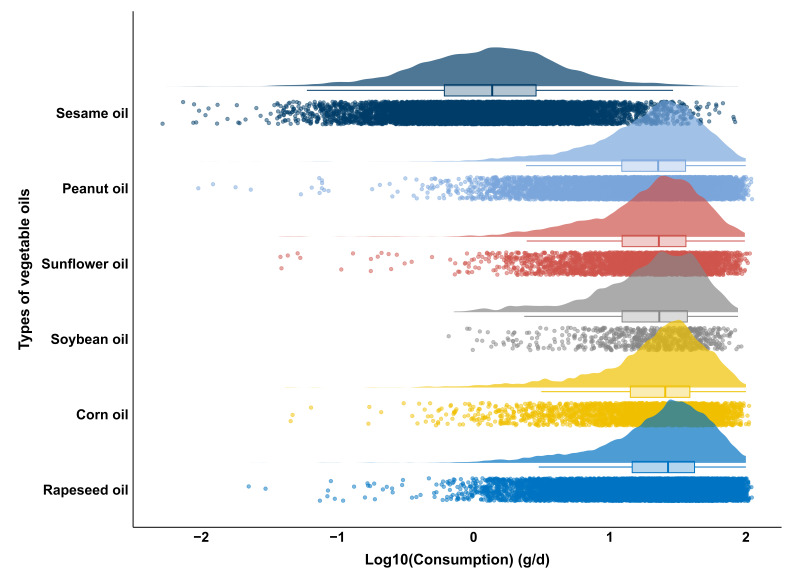
Distribution of dietary consumption for different types of vegetable oils (consumption values on log10-scale). Each panel shows individual consumption points (dots), the corresponding boxplot (median and interquartile range), and a violin curve representing the probability density of consumption levels. The width of the violin indicates the frequency of individuals at a given consumption level.

Data from the 2017–2020 China Total Diet Study [20] provided vegetable oil consumption data for 37,649 individuals. Oils were categorized into six types: peanut, rapeseed, soybean, corn, sesame, and sunflower oil. Across all oil types, the proportion of female consumers (51.6%) was slightly higher than males (48.4%). Overall, vegetable oil consumption among the Chinese population was mainly concentrated at low to moderate levels, with a smaller proportion of individuals exhibiting higher intake. The mean intake was 27.35 g/d, the median 23.91 g/d, and the 95th percentiles reached 67.23 g/d, suggesting potential high-exposure scenarios for a small portion of the population. Consumption patterns varied among oil types. Rapeseed, peanut, and corn oils were the most commonly consumed, with median intakes exceeding 25 g/d and 95th percentiles exceeding 62 g/d, representing the primary sources of dietary oils. In contrast, sesame oil showed a comparatively low average consumption (2.74 g/d), though occasional extreme consumption was observed (maximum 87.71 g/d), reflecting distinct regional dietary habits. Results are shown in Figure 3 and Table S5.

### 3.4. Probability Assessment of Exposure to SCCPs and MCCPs in Vegetable Oils

**Figure 4:**
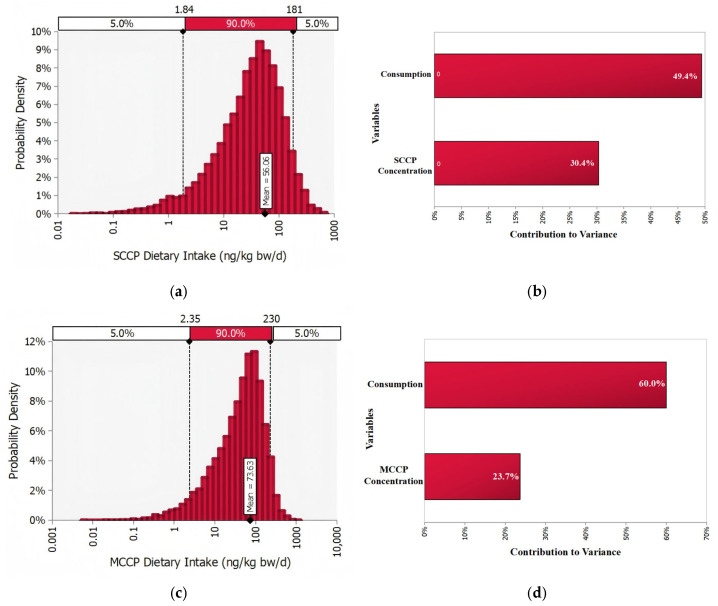
Probabilistic distributions of dietary exposure and MOE for SCCPs and MCCPs (MOE values on log10-scale). (**a**) Probabilistic distribution of dietary exposure to SCCPs; (**b**) Sensitivity analysis of SCCPs exposure based on Contribution to Variance; (**c**) Probabilistic distribution of dietary exposure to MCCPs; (**d**) Sensitivity analysis of MCCPs exposure based on Contribution to Variance.

The probabilistic assessment (Figure 4) revealed that daily exposure to SCCPs in vegetable oils followed a right-skewed distribution, with a mean of 56.06 ng/kg bw/d, median of 35.29 ng/kg bw/d, and P95 of 180.91 ng/kg bw/d. Corresponding MOE were 4.10 × 10^4^, 6.52 × 10^4^, and 1.27 × 10^4^, respectively. Similarly, MCCPs exposure exhibited a right-skewed distribution with a mean of 73.63 ng/kg bw/d, median of 49.76 ng/kg bw/d, and P95 of 230.49 ng/kg bw/d, corresponding MOE of 4.89 × 10^5^, 7.23 × 10^5^, and 1.56 × 10^5^. All MOE exceeded 1000, indicating a low potential health risk for both the general population and high consumption groups. Using the Contribution to Variance metric in @RISK, sensitivity analysis showed that dietary exposure variability was mainly driven by oil consumption (49.4% for SCCPs and 60.0% for MCCPs), followed by contaminant concentrations (30.4% and 23.7%, respectively). These results indicate that consumption amount is the primary determinant of overall exposure.

## 4. Discussion

In recent years, the extensive production and use of CPs have led to their widespread detection in various environmental and biological samples. In particular, the detection frequency of SCCPs and MCCPs in humans has increased rapidly [13,24]. Growing evidence suggests that CPs are bioaccumulative, and long-term exposure may induce oxidative stress, lipid metabolism disorders, and endocrine disruption, thereby posing potential health risks to humans. Consequently, their health effects have received increasing global attention [25,26,27]. Dietary intake serves as the primary exposure route. A recent study found that dietary intake accounted for approximately 88% and 93% of total SCCPs and MCCPs exposure among adults in Beijing, respectively [6]. Moreover, plant-based foods generally contain higher CPs levels, with edible vegetable oils showing significantly greater concentrations than other food categories [28].

The concentration data revealed considerable variation in SCCPs and MCCPs levels among different oil types. The mean SCCP concentrations in this study were higher than those reported in the Netherlands [29] (<8–78 ng/g lw; mean: 12 ng/g lw) and other European markets [11,30]. Similarly, the levels exceeded those observed by Cao et al. in Shanghai and Shenyang (<9–240 ng/g, median: <9 ng/g; and <20–210 ng/g, median: <20 ng/g, respectively) [8]. However, they remained lower than those measured in Beijing [31] (18–1100 ng/g, median: 520 ng/g) and in samples exported to Japan (<9–7500 ng/g, median: 94 ng/g). Among the six types analyzed, sunflower, peanut, sesame, and rapeseed oils showed SCCP concentrations higher than those reported by Shen et al. for Dutch oils (mean: <8, 31, 13, and <8 ng/g, respectively), yet lower than those reported by Gao et al. (2020) for the same types from China (mean: 217, 385, 1364, and 1252 ng/g, respectively) [15]. In contrast, concentrations in soybean and corn were comparable to those reported by Gao et al. Collectively, these results suggest that SCCPs contamination in Chinese oils remains significant. Details are provided in Table S6.

The MCCP concentrations in the analyzed vegetable oils were higher than those reported in the Netherlands and Belgium (<7–391 ng/g lw, mean: 69 ng/g lw and <12–190 ng/g, respectively) [29], but significantly lower than the levels measured in vegetable oil samples from Chinese supermarkets (<3–12,769 ng/g) [15]. The average concentrations in sunflower, peanut, sesame, and rapeseed oils were also higher than those reported in the Netherlands (21, 120, 72, and 24 ng/g, respectively) [29]. Except for soybean oil, the MCCPs levels in the remaining oil types were lower than those reported in China by Gao et al. (2020) [15], yet remained higher than European values, likely due to earlier CPs regulatory measures in Europe. Overall, these findings indicate that MCCPs contamination in Chinese edible vegetable oils remains a concern. Details are provided in Table S7.

SCCP and MCCP concentrations varied across edible oil types, with MCCPs exceeding SCCPs in most samples, contrary to previous reports on CPs in Chinese vegetable oils. This difference is likely related to the regulatory restrictions on SCCPs. Following the inclusion of SCCPs in Annex A of the Stockholm Convention in 2017 [16], China implemented corresponding measures. This involved listing SCCPs in the Catalogue of Priority Controlled Chemicals (First Batch) [32] and the List of Key Controlled New Pollutants (2023 Edition) [17], which prohibits their production, processing, use, import and export from 1 January 2024. Before these regulatory actions, SCCPs predominated in domestic polymeric products such as plastics, rubber, and food packaging in China [33], whereas more recent surveys indicate that MCCPs now occur at substantially higher levels in food packaging materials [34]. With the progressive strengthening of these regulations, the mean SCCP concentration in this study (112 ng/g) was markedly lower than previously reported values (ND-16,055 ng/g) [8,15] in edible vegetable oils. This decline likely reflects the combined effects of enhanced regulatory actions and gradual industrial substitution. However, the predominance of MCCPs highlights the need for continued monitoring and control. Spearman correlation analysis further revealed a significant positive correlation between SCCP and MCCP concentrations (r = 0.411, *p* < 0.05), suggesting that they may originate from similar contamination sources. In addition, SCCPs volatilize more readily than MCCPs during refining. As most Chinese vegetable oils produced by pressing or solvent extraction undergo refining, this process may also contribute to the relatively higher MCCPs levels.

The dietary exposure of the Chinese population to SCCPs and MCCPs is strongly associated with edible oil consumption. Previous studies have shown that diet is the primary exposure route for CPs, accounting for more than 50% of total exposure [6], with edible oils exhibiting substantially higher SCCP concentrations than other food categories [9]. As the world’s largest consumer of vegetable oils, China’s staple oils, such as soybean and rapeseed, are the main contributors to population exposure owing to their high consumption [35]. However, our results indicate that consumption volume is not the sole determinant of risk. Low-consumption oils, such as sesame oil, exhibited high contamination levels, which may result in exposure levels in certain consumer groups comparable to those from high-consumption oils.

The integration of probabilistic assessment and Monte Carlo simulation enhances the robustness and representativeness of exposure estimation. Considering the right-skewed distribution of edible oil consumption in the Chinese population and the variation in contamination levels among oil types (e.g., bulk oils vs. sesame oil), the model was designed to capture both variability and uncertainty. Probability density functions were assigned to each input parameter (e.g., consumption levels and contaminant concentrations) within the Monte Carlo framework. The resulting exposure distributions illustrate the exposure and risk profiles across general and high-consumption populations, providing a more realistic reflection of actual SCCPs and MCCPs exposures than a single conservative estimate.

Notably, although dietary exposure levels of SCCPs and MCCPs vary across countries and regions, the corresponding MOE consistently exceed 1000, suggesting a low potential health risk under current exposure scenarios. For example, a study from Jinan, China, reported an SCCPs exposure of 3109 ng/kg bw/d [36], substantially higher than that estimated in the present work, likely due to elevated local contamination. Another study demonstrated that cooking can markedly reduce SCCPs intake, from 403 ng/kg bw/d in raw foods to 145 ng/kg bw/d in cooked ones [37]. By comparison, exposures reported in Japan [36] (17.8 ng/kg bw/d) and Germany (160 ng/kg bw/d) were both lower than those in the present study, reflecting regional differences in contamination levels and dietary patterns. Interestingly, the German data were comparable to the MCCPs exposure estimated in this study, underscoring the combined influence of environmental contamination and dietary habits on overall CPs intake. Consistent with these observations, the estimated dietary exposure to SCCPs and MCCPs in the present study also indicated no apparent adverse health effects, as all MOE values exceeded the threshold of 1000.

However, this study has several limitations and uncertainties. The probabilistic assessment model provides a more comprehensive characterization of variability in contaminant concentrations and population consumption patterns. However, the reliability of the outcomes largely depends on the assumed probability distributions of input parameters, introducing methodological uncertainty. Regarding concentration data, although the collected samples covered major production regions and commonly consumed oil types across China, their representativeness may still be limited. Despite rigorous analytical quality assurance and control, the complex mixture of SCCPs and MCCPs congeners, matrix interferences, lack of certified reference materials, and differences in instrumental responses may have affected measurement accuracy and precision, thereby increasing uncertainty in exposure estimation. Moreover, this study focused exclusively on dietary exposure through edible vegetable oils, without accounting for other potential pathways such as inhalation, drinking water, other food categories (e.g., meat and aquatic products), or dermal contact. Consequently, the cumulative exposure and associated health risks of chlorinated paraffins may have been underestimated.

## 5. Conclusions

This study provides the first probabilistic exposure assessment of CPs in Chinese edible vegetable oils, offering current contamination profiles. Clear differences in contamination levels were observed across oil types, with SCCPs highest in sesame oil and MCCPs more elevated in peanut and soybean oils. Overall, MCCP concentrations were higher than those of SCCPs, suggesting that the SCCPs ban is already influencing market patterns. Although the estimated dietary exposure to SCCPs and MCCPs through vegetable oils indicated no apparent adverse health effects based on the MOE values, sesame, peanut, and soybean oils should be prioritized in future monitoring programs. At the same time, the food industry should reinforce quality control across processing and packaging to further minimize contamination. It should be noted that other dietary sources and other exposure routes such as dermal uptake should not be neglected. Moreover, considering the persistent, bioaccumulative potential, and widespread application of CPs, future work should adopt multimedia and multipathway cumulative exposure assessments to better elucidate their long-term health implications for the Chinese population.

## Data Availability

The original contributions presented in this study are included in the article/Supplementary Materials. Further inquiries can be directed to the corresponding authors.

## Supplementary Materials

The following supporting information can be downloaded at: https://www.mdpi.com/article/10.3390/foods14233988/s1, Text S1. Detailed extraction and cleanup procedures for edible oil samples. Figure S1. A Total Ion Chromatogram (TIC) of a Vegetable Oil Sample. Figure S2. ECNI-MS spectrum of C14Cl7 (MCCP). Figure S3. ECNI-MS spectrum of C10Cl6 (SCCP). Table S1. Specific information and concentration (ng/g) of SCCPs and MCCPs in different types of vegetable oil samples. Table S2. Concentrations of SCCPs and MCCPs in Different Vegetable Oils (ng/g). Table S3. The concentrations of SCCP congener group (ng/g) in different types of vegetable oil. Table S4. The concentrations of MCCPs congener group (ng/g) in different types of vegetable oil. Table S5. Daily consumption of edible vegetable oils (g/d) in the Chinese population (2017–2020). Table S6. Concentrations of SCCPs in Different Countries (ng/g wet weight). Table S7. Concentrations of MCCPs in Different Countries (ng/g wet weight). Reference [38] is cited in Supplementary Materials.
