# Supplementary material for: Occurrence, Homologue Profiles and Risk Assessment of Short- and Medium-Chain Chlorinated Paraffins in Edible Vegetable Oils
Source: Foods. 2025 Nov 21;14(23):3988. doi: 10.3390/foods14233988 (PMC12691766; doi:10.3390/foods14233988)
Supplement: Supplementary file 1 [file foods-14-03988-s001.zip › foods-3954893-supplementary.pdf]

Supplementary Materials

Text S1. Detailed extraction and cleanup procedures for edible oil samples

2.00 g of vegetable oil was extracted with 70 mL n-hexane/dichloromethane (1:1, v/v) and spiked with 2.5 ng 13C10-trans-chlordane. The extract was first purified using a gel permeation chromatography (GPC) column to remove sulfur-containing compounds, lipids, and other interferences. After sample loading, the column was eluted with 70 mL of dichloromethane/n-hexane (1:1, v/v) to discard the initial eluate, followed by 130 mL of the same solvent mixture to collect the target fraction, which was then concentrated to approximately 2 mL. The extract was further purified on a multilayer silica column composed of 3 g Florisil, 2 g activated silica gel, 5 g acidified silica gel (44%, w/w), and 4 g anhydrous sodium sulfate. The column was pre-rinsed with 50 mL n-hexane before sample loading. The sample was eluted with 40 mL n-hexane (discarded) and 100 mL dichloromethane/n-hexane (1:1, v/v) to collect the target compounds. The eluate was concentrated to about 2 mL using rotary evaporation and transferred into a KD concentrator. The solution was further reduced to approximately 0.5 mL under a gentle nitrogen stream and solvent-exchanged to 50 μL cyclohexane containing 2.5 ng ε-hexachlorocyclohexane as an internal standard.

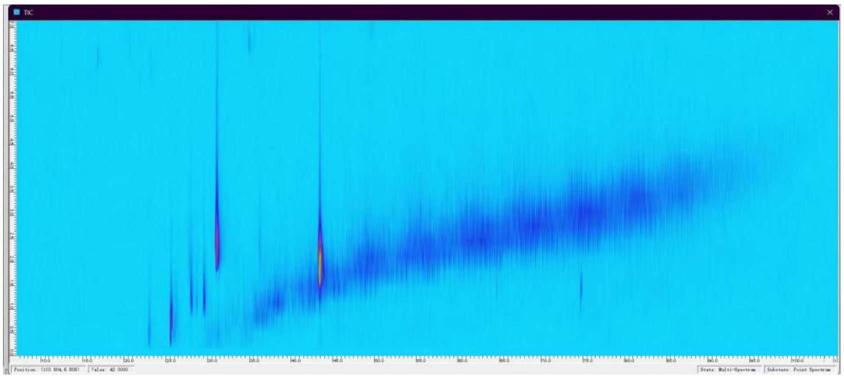

Figure S1 A Total Ion Chromatogram (TIC) of a Vegetable Oil Sample

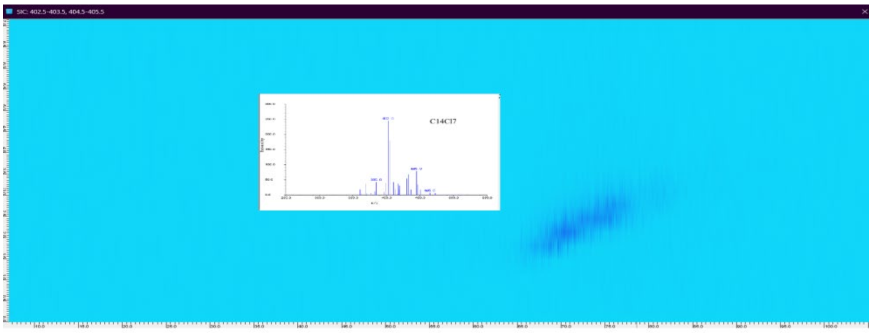

Figure S2 ECNI-MS spectrum of C14Cl7 (MCCP)

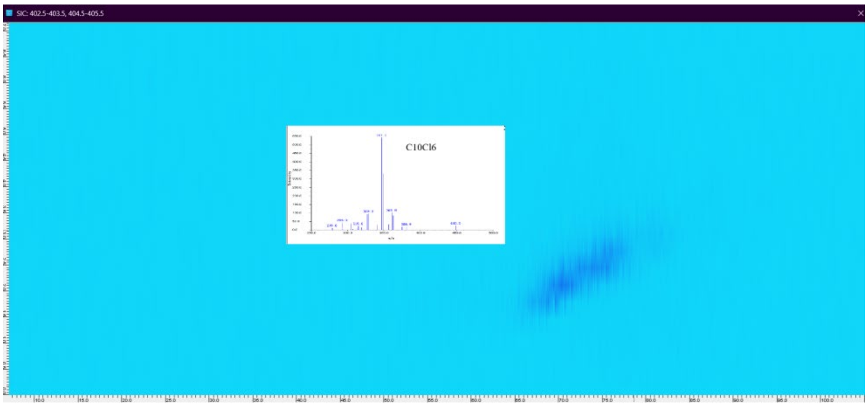

Figure S3 ECNI-MS spectrum of C10Cl6 (SCCP)

Table S1 Specific information and concentration（ng/g）of SCCPs and MCCPs in different types of vegetable oil samples

Code	Type	Brand	Packaging	Origin	Process	SCCPs	MCCPs
S1	Sunflower oil	Jinlongyu	Plastic	Qinhuangdao	Pressed	ND	17
S2	Sunflower oil	Xiwang	Plastic	Binzhou	Pressed	86	83
S3	Sunflower oil	Jiage	Plastic	-	Pressed	34	40
S4	Sunflower oil	Luhua	Plastic	-	Pressed	41	47
S5	Peanut oil	Hujihua	Plastic	Shijiazhuang	Pressed	174	200
S6	Peanut oil	Luhua	Plastic	-	Pressed	125	189
S7	Peanut oil	Longyuan	Plastic	-	Pressed	86	133
S8	Peanut oil	Jinlongyu	Plastic	Shijiazhuang	Pressed	120	341
S9	Peanut oil	Fulinmen	Plastic	Linyi	Pressed	101	191
S10	Soybean oil	Jiusan	Plastic	Harbin	Extracted	278	200
S11	Soybean oil	Fulinmen	Plastic	Tianjin	Extracted	38	122
S12	Soybean oil	Jinlongyu	Plastic	Qinhuangdao	-	48	226
S13	Soybean oil	Jiusan	Aluminum	Harbin	Pressed	119	182
S14	Soybean oil	Jiusan	Plastic	Harbin	Extracted	114	205
S15	Sesame oil	Lee Kum Kee	Glass	Jiangmen	Pressed	283	126
S16	Sesame oil	Jinlongyu	Glass	-	-	127	85
S17	Sesame oil	Luhua	Glass	-	-	55	31
S18	Sesame oil	Qianhe	Glass	Meishan	Pressed	219	150
S19	Sesame oil	Fulinmen	Glass	Hefei	Pressed	142	19
S20	Rapeseed	Daodaoquan	Plastic	-	Pressed	166	257

	oil						
S21	Rapeseed oil	Fulinmen	Plastic	Jingmen	Pressed	110	126
S22	Rapeseed oil	Luhua	Plastic	-	Pressed	98	128
S23	Corn oil	Changshouhua	Plastic	Binzhou	Pressed	ND	106
S24	Corn oil	Shanrun	Plastic	Yueyang	Pressed	109	192
S25	Corn oil	Fulinmen	Plastic	Tianjin	Pressed	51	130
S26	Corn oil	Xiwang	Plastic	Zouping	Pressed	62	108
S27	Corn oil	Jinlongyu	Plastic	Qinhuangdao	Pressed	71	131
S28	Corn oil	Jinlongyu	Plastic	Zouping	Pressed	87	118
S29	Corn oil	Xiwang	Plastic	Qinhuangdao	Pressed	96	141

-, not recorded

Table S2. Concentrations of SCCPs and MCCPs in Different Vegetable Oils (ng/g).

type of oils	N	SCCPs			MCCPs		
		Average	Min	Max	Average	Min	Max
sunflower oil	4	54	ND	86	47	17	83
peanut oil	5	121	86	125	211	133	341
soybean oil	5	119	38	278	187	122	226
sesame oil	5	165	55	283	82	19	150
rapeseed oil	3	125	98	166	170	126	257
corn oil	7	79	ND	109	132	106	192
Total	29	112	ND	283	139	17	341

ND, not detected.

Table S3. The concentrations of SCCP congener group (ng/g) in different types of vegetable oil.

SCCPs	Sunflower oil	Peanut oil	Soybean oil	sesame oil	Rapeseed oil	Corn oil
10,5	2	3.2	3.3	8.2	2.7	2.3
10,6	7.2	13.2	16.1	29.2	12.5	8.8
10,7	6.4	12.7	16.7	21.8	13.6	6.7
10,8	2.3	5.7	6.7	7.3	6.8	2.6
10,9	0.5	1.4	1.5	1.3	1.7	0.7
10,10	0.2	0.4	0.4	0.3	0.5	0.3
**$\sum C_{10}$**	**18.5**	**36.6**	**44.7**	**68**	**37.7**	**21.3**
11,5	1	1.7	1.6	3.5	1.3	1.3
11,6	3.2	6.7	7.7	12.1	7.2	3.6
11,7	4.9	10.2	10.9	16	11.7	5.9
11,8	3	7.6	6.7	10	8.9	4.9
11,9	0.9	2.5	2.1	2.6	3.1	1.4
11,10	0.3	0.7	0.9	0.6	0.8	0.5

**∑C_{11}**	**13.1**	**29.4**	**29.9**	**44.8**	**33.1**	**17.6**
12,5	1.3	1.7	1.9	2.3	1.5	1.9
12,6	1.8	3.1	4.2	4.7	3.6	2.2
12,7	3.5	7.1	7.2	8.7	7.9	5.8
12,8	2.7	7.8	6	7.3	8.4	5.8
12,9	1	3.4	2.4	2.6	4	2.3
12,10	0.3	1.1	1	0.7	1.3	0.6
**∑C_{12}**	**10.8**	**24.2**	**22.7**	**26.2**	**26.6**	**18.5**
13,5	0.5	0.8	0.8	1	0.8	0.7
13,6	1.6	3.3	3.1	3.7	3.2	2.8
13,7	4.4	10.5	7.9	9.6	9.4	9.2
13,8	3.2	10.4	6.5	7.9	8.7	8.3
13,9	1.2	4.8	2.7	3.3	4	3.4
13,10	0.4	1.4	1	0.8	1.3	0.8
**∑C_{13}**	**11.3**	**31.1**	**22**	**26.3**	**27.3**	**25.1**
**∑SCCPs**	**53.7**	**121.3**	**119.2**	**165.3**	**124.7**	**82.4**

Table S4. The concentrations of MCCPs congener group (ng/g) in different types of vegetable oil.

MCCPs	Sunflower oil	Peanut oil	Soybean oil	sesame oil	Rapeseed oil	Corn oil
14,5	1.4	3.2	3	2.4	3.9	2.3
14,6	3.9	14.1	14.4	8.1	16.6	6.5
14,7	1.3	3	2.5	2.4	3	9.8
14,8	7.5	35.8	33.5	15.6	35.1	17.5
14,9	2	7.2	6	3.7	6.6	6.2
14,10	1.6	4.2	3.7	2.9	4.5	2.6
**∑C_{14}**	**17.6**	**67.5**	**63.1**	**35.1**	**69.8**	**44.9**
15,5	7.1	37.1	32.9	12.1	27	10.7
15,6	3.2	16	13.8	6.1	14.2	7.4
15,7	1.4	4.1	4	2.4	3.7	6.7
15,8	1.5	3.4	3.1	2.6	8.9	7.9
15,9	3.2	16.9	14	3.9	8	6.7
15,10	3.1	18.5	15.6	5.4	12.5	6.6
**∑C_{15}**	**19.5**	**96**	**83.4**	**32.5**	**74.4**	**45.9**
16,5	1.8	7	7.2	3.1	5.5	5.1
16,6	1	2.9	2.3	1.6	2.5	3.3
16,7	0.8	4.1	3.4	0.8	1.5	4.2
16,8	1.4	8.9	7.7	2.1	4.3	6.2
16,9	1.4	8.2	7.3	2.3	4.7	5.2
16,10	0.9	3.5	2.6	1.4	2.4	2.4
**∑C_{16}**	**7.3**	**34.6**	**30.5**	**11.2**	**20.9**	**26.3**
17,5	0.4	2.1	1.9	0.5	0.8	2.9

17,6	0.7	4	3.5	0.9	1.8	3.2
17,7	0.7	3.7	2.4	1	1.7	4.2
17,8	0.2	0.8	0.8	0.3	0.4	2.3
17,9	0.3	1.5	1	0.4	0.6	1.5
17,10	0.1	0.2	0.2	0.1	0.1	0.2
**$\sum C_{17}$**	**2.4**	**12.4**	**9.8**	**3.2**	**5.3**	**14.2**
**∑MCCPs**	**47**	**211**	**187**	**82**	**170**	**187.3**

Table S5. Daily consumption of edible vegetable oils (g/d) in the Chinese population (2017–2020).

Oil Type	Sample Size (n)	Mean	P50	P95	Minimum	Maximum
Rapeseed Oil	15354	30.03	26.62	70.11	0.02	99.90
Peanut Oil	12142	26.18	22.64	62.80	0.01	99.71
Sesame Oil	9791	2.74	1.37	9.24	0.01	87.71
Sunflower Oil	3801	22.64	18.64	62.86	0.01	100.00
Corn Oil	3699	28.54	25.49	66.60	0.04	100.00
Soybean Oil	707	26.07	23.13	59.47	0.72	87.47
Total	37649	27.35	23.91	67.23	0.01	183.76

Table S6. Concentrations of SCCPs in Different Countries (ng/g wet weight)

Country	Region	N	Type	Range	mean	P50	Ref.
China	Shanghai	6	Peanut, maize, rapeseed, soybean, sunflower seed oils	<9-240	-	<9	[1]
	Beijing	7	Soybean, mustard, sesame seed, olive oils	18–1100	-	520	[1]
	Shenyang	6	Peanut, maize, soybean, sunflower seed, mixed oils	<20–210	-	<20	[1]
	Hong Kong	5	Peanut, maize, olive oils	<9–230	-	170	[1]
	-	16	rapeseed oil	ND-12769	270	292	[2]
	-	10	camellia oil	69-1260	319	249	[2]
	-	22	sesame oil	21.8-8429	329	244	[2]
	-	20	peanut oil	13.5-2769	201	207	[2]
	-	5	linseed oil	22.2-1337	223	168	[2]
	-	18	blended oil	ND-16055	218	115	[2]
	-	6	rice oil	ND-230	19.4	66.7	[2]
	-	13	corn oil	2.06-781	40.1	34.8	[2]
	-	7	soybean oil	ND-522	57.4	31.9	[2]
	-	41	olive oil	ND-3157	16.5	17.7	[2]
	-	5	sunflower seed oil	2.5-1007	23	8.25	[2]
Japan	Yokoh	9	Peanut, sesame seed, pepper	<9–7500	-	94	[1]

	ama		oils				
Netherlands	-	15	Olive oil	<8-78	14	-	[3]
	-	6	Sunflower oil	<8-<8	<8	-	[3]
	-	3	Peanut oil	13-63	31	-	[3]
	-	2	Sesame oil	10-17	13	-	[3]
	-	2	Linseed oil	<8-<8	<8	-	[3]
	-	2	Grapeseed oil	<8-16	10	-	[3]
	-	2	Rapeseed oil	<8-<8	<8	-	[3]
	-	2	Rice bran oil	<8-<8	<8	-	[3]
	-	5	Other vegetable oils	<8-31	13	-	[3]
	-	39	Sum (all oils)	<8-78	12	-	[3]
Germany	-	16	olive	-	44	-	[4]
	-	18	rapeseed	-	13	-	[4]
Czech Republic	-	1	Rapeseed oil	<10	-	<10	[5]
	-	1	Rapeseed oil	<10	-	<10	[5]
	-	1	Rapeseed oil	<10	-	<10	[5]
	-	1	Rapeseed oil	<10	-	<10	[5]
	-	1	Rapeseed oil	<10	-	<10	[5]
	-	1	Sunflower oil	<20	-	<20	[5]
	-	1	Sunflower oil	<20	-	<20	[5]
Belgium	-	26	oil	ND-19	-	ND	[6]

-, not recorded; ND, not detected. Concentrations converted from lipid weight (lw) to wet weight (ww) based on reported lipid content.

Table S7. Concentrations of MCCPs in Different Countries (ng/g wet weight)

Country	Region	N	Type	Range	mean	P50	Referencr
China	-	16	rapeseed oil	ND-12769	130	220	[2]
	-	10	camellia oil	68.7-547	247	328	[2]
	-	22	sesame oil	ND-6210	53.3	81.7	[2]
	-	20	peanut oil	ND-3667	110	148	[2]
	-	5	linseed oil	5.97-1243	90.6	97.6	[2]
	-	18	blended oil	ND-11612	94.9	109	[2]
	-	6	rice oil	ND-ND	11.8	34.8	[2]
	-	13	corn oil	ND-284	26.4	68.2	[2]
	-	7	soybean oil	ND-1018	160	153	[2]
	-	41	olive oil	ND-ND	4.39	49	[2]
	-	5	sunflower seed oil	ND-756	19.2	65.3	[2]
Netherlands	-	15	Olive oil	<7-266	44	-	[3]
	-	6	Sunflower oil	<7-55	21	-	[3]
	-	3	Peanut oil	120-279	210	-	[3]
	-	2	Sesame oil	49-94	72	-	[3]

	-	2	Linseed oil	8-22	15	-	[3]
	-	2	Grapeseed oil	11-86	48	-	[3]
	-	2	Rapeseed oil	16-32	24	-	[3]
	-	2	Rice bran oil	384-391	388	-	[3]
	-	5	Other vegetable oilsc	10-72	39	-	[3]
	-	39	Sum (all oils)	<7-391	69	-	[3]
Germany	-	18	olive		300	-	[4]
	-	16	rapeseed		18	-	[4]
Czech Republic	-	1	Rapeseed oil 1	-	<20	-	[5]
	-	1	Rapeseed oil 2	-	32 ± 8	-	[5]
	-	1	Rapeseed oil 3	-	<20	-	[5]
	-	1	Rapeseed oil 4	-	<20	-	[5]
	-	1	Rapeseed oil 5	-	<20	-	[5]
	-	1	Sunflower oil 6	-	<20	-	[5]
	-	1	Sunflower oil 7	-	<20	-	[5]
Belgium	-	26	oil	ND-190	-	40	[6]

-, not recorded; ND, not detected. Concentrations converted from lipid weight (lw) to wet weight (ww) based on reported lipid content.

[1] Cao Y, Harada KH, Liu W, et al. Short-chain chlorinated paraffins in cooking oil and related products from China. Chemosphere. 2015;138:104-111. doi:10.1016/j.chemosphere.2015.05.063

[2] Gao W, Bai L, Ke R, et al. Distributions and Congener Group Profiles of Short-Chain and Medium-Chain Chlorinated Paraffins in Cooking Oils in Chinese Markets. J Agric Food Chem. 2020;68(29):7601-7608. doi:10.1021/acs.jafc.0c02328

[3] Shen Y, Krätschmer K, Bovee T, et al. Chlorinated paraffins (CPs) in vegetable oils from the Dutch market and the effects of the refining process on their levels[J]. Food Control, 2023, 153: 109889.

[4] Krätschmer K, Schächtele A, Vetter W. Short- and medium-chain chlorinated paraffin exposure in South Germany: A total diet, meal and market basket study. Environ Pollut. 2021;272:116019. doi:10.1016/j.envpol.2020.116019

[5] Tomasko J, Stupak M, Hajslova J, Pulkrabova J. Application of the GC-HRMS based method for monitoring of short- and medium-chain chlorinated paraffins in vegetable oils and fish. Food Chem. 2021;355:129640. doi:10.1016/j.foodchem.2021.129640

[6] McGrath T J, Limonier F, Poma G, et al. Concentrations and distribution of chlorinated paraffins in Belgian foods[J]. Environmental Pollution, 2021, 291: 118236.
